# Impact of HIV/AIDS on care and outcomes of severe sepsis

**DOI:** 10.1186/cc3811

**Published:** 2005-09-27

**Authors:** Joseph M Mrus, LeeAnn Braun, Michael S Yi, Walter T Linde-Zwirble, Joseph A Johnston

**Affiliations:** 1 Research Physician, Health Services Research and Development, Cincinnati VA Medical Center, Cincinnati, OH, USA; 2 Assistant Professor, Department of Internal Medicine and Institute for the Study of Health, University of Cincinnati Medical Center, Cincinnati, OH, USA; 3 Manager, Clinical Development, Infectious Diseases Medicine Development Center – HIV, GlaxoSmithKline, Research Triangle Park, NC, USA; 4 Associate Clinical Development Consultant, Corporate Clinical Operations, Eli Lilly and Company, Indianapolis, IN, USA; 5 Assistant Professor, Department of Internal Medicine and Institute for the Study of Health, University of Cincinnati Medical Center, Cincinnati, OH, USA; 6 Vice President, Chief Science Officer, ZD Associates, LLC, Perkasie, PA, USA; 7 Clinical Research Physician, US Outcomes Research, Lilly Research Laboratories, Indianapolis, IN, USA

## Abstract

**Introduction:**

There has been dramatic improvement in survival for patients with HIV/AIDS; however, some studies on patients with HIV/AIDS and serious illness have reported continued low rates of intensive care. The purpose of this study was to examine patterns of care and outcomes for patients with severe sepsis and HIV/AIDS and compare them with those of patients with severe sepsis without HIV/AIDS.

**Methods:**

We assessed data from all 1999 discharge abstracts from all non-federal hospitals in six US states. Patient demographic characteristics, discharge diagnoses, resource use, and outcomes were extracted. Analyses were performed using chi-square, Wilcoxon rank sum, or regression techniques, as appropriate.

**Results:**

We identified 74,020 patients with severe sepsis (7,638 (10.3%) had HIV/AIDS) using ICD-9-CM codes. Patients with severe sepsis and HIV/AIDS had a similar mean length of stay (16.9 days versus 17.7 days; p = 0.0669), had lower mean hospitalization cost ($24,382 versus $30,537; p < 0.0001), were less likely to be admitted to the intensive care unit (37% versus 56%; p < 0.0001), and had a greater mortality (29% versus 20%; p < 0.0001) than those without HIV/AIDS. After adjustment for cohort differences, patients with severe sepsis and HIV/AIDS had increased likelihood of death (OR (95% CI) = 2.41 (2.23–2.61)) and were substantially less likely to be admitted to the intensive care unit (OR (95% CI) = 0.54 (0.51–0.59)). When compared with those with severe sepsis and HIV/AIDS, patients with severe sepsis without HIV/AIDS were universally more likely to be admitted to the intensive care unit, even when they had comorbid illnesses with equal or worse expected in-hospital mortality (e.g., metastatic cancer).

**Conclusion:**

For patients with severe sepsis, there are differences in care and outcomes for those with HIV/AIDS. Further research is needed to examine the delivery of care for patients with severe sepsis and HIV/AIDS.

## Introduction

With the advent of highly active antiretroviral therapy (HAART) in the late 1990s, opportunistic infection and mortality rates for patients with HIV/AIDS have dramatically decreased, thus transforming HIV/AIDS from a uniformly fatal condition to a more manageable chronic illness [1-5]. Improvement in care and survival have also extended to HIV/AIDS patients with severe infections and those who receive care in the intensive care unit (ICU) [6-9]. While studies have shown dramatic improvement in survival related to intensive care for patients with HIV/AIDS in the HAART era, some studies in patients with HIV/AIDS and serious illness have reported continued low rates of intensive care [9,10].

In 1992, the American College of Chest Physicians/Society of Critical Care Medicine Consensus Conference arrived at the current definition of severe sepsis (SS) as a systemic inflammatory syndrome in response to infection that is associated with acute organ dysfunction [11]. Subsequent studies have shown that SS results in substantial morbidity and mortality for all patients, especially for patients with comorbid illnesses, including HIV/AIDS [12-14]. However, those data pre-date the HAART era, and there are few data directly comparing outcomes and resource use for patients with SS and HIV/AIDS versus patients with SS but without HIV/AIDS. Thus, the purpose of this study was two-fold: to examine patterns of care and outcomes for patients with SS and HIV/AIDS; and to assess differences in patterns of care and outcomes for those with SS and HIV/AIDS versus those with SS without HIV/AIDS.

## Materials and methods

### Data sources

Data from discharge abstracts for calendar year 1999 from all non-federal hospitals from six US states (Florida, Massachusetts, New Jersey, New York, Virginia, and Washington) were assessed. We selected those states based on geographic representation, data quality and availability. Data extracted included: patient demographic characteristics; diagnoses and procedures (principal discharge diagnosis, up to 14 secondary discharge diagnoses, and hospital procedures) classified by the International Classification of Diseases, 9th revision, Clinical Modification (ICD-9-CM) codes; resource use (hospital length of stay (LOS), ICU use, total charges); and in-hospital mortality.

### Case definition

Because no ICD-9-CM code existed at the time these data were collected that directly identified cases of severe sepsis, we identified cases by using an algorithm described by Angus and colleagues [13] and adapted by others [14,15] that required ICD-9-CM codes for a bacterial or fungal infection in addition to acute organ dysfunction. HIV/AIDS cases were identified using ICD-9-CM codes (042, V08) as outlined in the Centers for Disease Control and Prevention coding guidelines [16,17]. To improve comparability between the HIV infected and uninfected groups, we excluded patients who were younger than 20 years, were older than 64 years, or had pregnancy-related hospitalizations.

### Covariate definitions

We defined a case as surgical if there was an ICD-9-CM code for an operating room procedure other than tracheostomy. Teaching hospital status was determined from the Health Care Financing Administration Provider Specific File [18]. Using classifications and methodology adapted from Deyo and associates [19], we grouped patients into one of 10 categories according to their pattern of chronic comorbid illnesses: no comorbidities, HIV/AIDS, diabetes, pulmonary disease, cardiovascular disease (old myocardial infarction, peripheral vascular disease, or late effects of cerebrovascular disease), renal disease, liver disease, neoplasm (malignancy or metastatic disease), multiple comorbidities without HIV/AIDS, and HIV/AIDS with at least one other comorbid illness. Respiratory infections were determined by selecting ICD-9-CM codes in the range 460–519, and opportunistic infections were determined by selecting appropriate ICD-9-CM codes as has been done by Keyes and coworkers [20] as well as others [21].

### Outcomes

Reported outcomes were ICU use (Medical ICUs, Surgical ICUs, or Coronary Care Units), hospital length of stay, total cost of the admission, and in-hospital mortality. We estimated the cost for each case by multiplying total charges by the sum of the hospital-specific Medicare capital and operating cost-to-charge ratios [18].

### Statistical analyses

The databases were constructed in Foxpro (Microsoft Corp., Redmond, WA, USA) and analyses were conducted using SAS version 8.2 (SAS Institute, Cary, NC, USA). We used chi-square or Fisher's exact test to compare categorical characteristics and Student's *t* test to compare continuous data. Odds ratios (ORs) were determined using simple regression. Adjusted analyses were performed using multivariable logistic or linear regression, as appropriate. All available covariates were included in the multivariable models. So that the results would be easily interpretable, interactions among variables were not pursued. The adjusted R^2^ or c-statistic is presented for each of the models. Although distributions for LOS and cost were not normally distributed, results were qualitatively similar whether analyses were performed with those values log transformed or not. Thus, we chose to not transform the data to facilitate interpretation.

To assess the robustness of our results, we performed additional stratified analyses. Specifically, additional analyses evaluating mortality and ICU admission were stratified by HIV/AIDS disease severity (presence of opportunistic infection or not) and additional outcomes comparisons were performed specifically with metastatic cancer (as opposed to all cancer) diagnoses. Also, because of the imbalance in characteristics between those with and without HIV, we were concerned about the robustness of our multivariable results. Thus, we performed additional analyses in subgroups with 'matched' characteristics. Specifically, we performed two additional analyses where we limited the cohort to patients aged 41 to 60 years without comorbidities (other than HIV for those with HIV infection) covered by Medicaid or Medicare who were admitted to a medical service in a teaching hospital. In the first analysis, we assessed only those admitted with respiratory infections but without opportunistic infections and compared outcomes for those with and without HIV infection. In the second analysis, we limited the analysis to only those admitted with opportunistic infections and compared outcomes for those with and without HIV.

## Results

We identified 74,020 cases of severe sepsis, 10.3% (n = 7,638) with HIV/AIDS (Table 1). Those with SS and HIV/AIDS were significantly younger on average (41.9 years versus 49.9 years); more likely to be male (66% versus 54%); less likely to be white (20% versus 56%); less likely to have commercial insurance (16% versus 42%); more likely to be admitted for medical reasons (88% versus 69%); more likely to be admitted at a teaching hospital (76% versus 61%); less likely to have comorbid illnesses (30% versus 51%); and more likely to have respiratory (45% versus 42%) and opportunistic infections (53% versus 9%) than those without HIV/AIDS (p ≤ 0.0001 for all comparisons; Table 1).

### Length of stay

**Figure 1:**
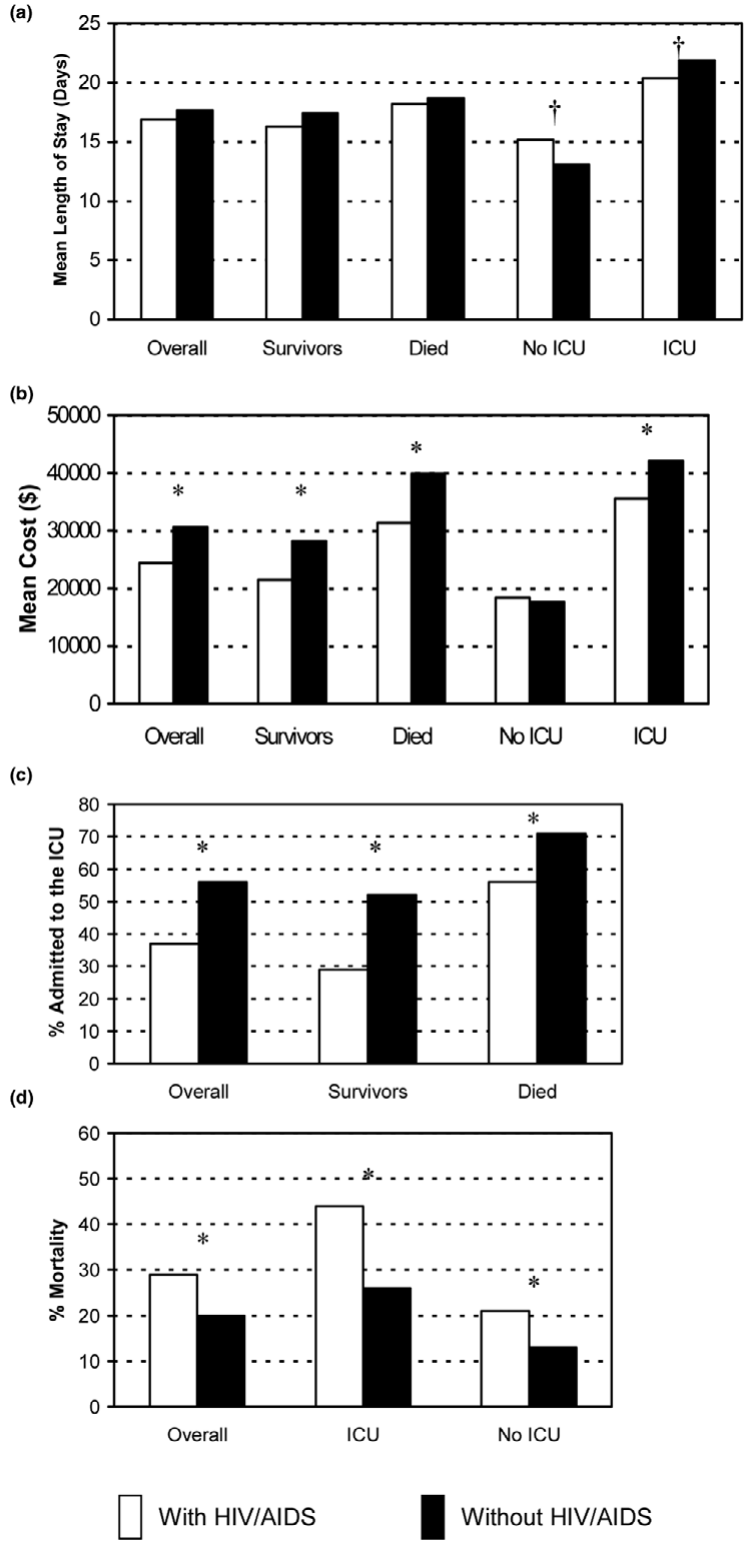
Pattern of care and outcomes for patients with severe sepsis with and without HIV/AIDS. **(a) **Mean length of stay, **(b) **mean hospitalization cost, **(c) **ICU admission rates, and **(d) **mortality rates are shown. Overall results, as well as results stratified by survival and intensive care unit (ICU) admission are shown (as appropriate). Patients with HIV/AIDS are denoted by the white bars and patients without HIV/AIDS by the black bars.*, p ≤ 0.0001; †, 0.0001 < p ≤ 0.05.

For patients with SS, those with HIV/AIDS had similar mean LOS (16.9 days) compared with those without HIV/AIDS (17.7 days; p = 0.0669; Fig. 1). There were no significant differences between those with and without HIV/AIDS when LOS results were stratified by mortality. However, the impact of HIV/AIDS on LOS varied by ICU admission status. For patients with SS not admitted to the ICU, those with HIV/AIDS had substantially longer LOS (15.2 days) than those without HIV/AIDS (13.1 days; p = 0.0028), and for patients with SS in the ICU, those with HIV/AIDS had substantially shorter LOS (20.4 days) than those without HIV/AIDS (21.9 days; p = 0.0005). After adjusting for differences in characteristics of patients with SS with and without HIV/AIDS through regression, those with HIV/AIDS did have a shorter LOS (-0.9 days); however, this difference was not statistically significant (p = 0.0516; Table 2).

### Hospitalization cost

A significantly lower mean hospitalization cost was observed for patients with SS and HIV/AIDS compared with those without HIV/AIDS ($24,382 versus $30,537; p < 0.0001; Fig. 1). The cost difference between patients with SS with and without HIV/AIDS remained significant even if results were stratified by mortality. However, the impact of HIV/AIDS on mean hospital cost varied by ICU admission status. For patients with SS not admitted to the ICU, those with HIV/AIDS incurred a similar mean cost ($18,495) to those without HIV/AIDS ($17,615; p = 0.0755); and for patients with SS in the ICU, those with HIV/AIDS incurred a significantly lower mean cost ($35,594) than those without HIV/AIDS ($42,111; p < 0.0001). After adjusting for cohort differences, the difference in hospitalization cost diminished from a difference of $6,155 to $2,706; however the difference remained statistically significant (p < 0.0001; Table 2).

### Intensive care unit admission and mortality

In patients with SS, those with HIV/AIDS were significantly less likely than those without HIV/AIDS to be admitted to the ICU (37% versus 56%; p < 0.0001) despite a statistically significant greater overall mortality (29% versus 20%; p < 0.0001; Fig. 1). In patients with SS, those with HIV/AIDS had significantly greater risk of death compared with those without HIV/AIDS whether or not they were admitted to the ICU (p < 0.0001). Regardless of whether patients survived, patients with HIV/AIDS were significantly less likely to have been admitted to the ICU than those without HIV/AIDS (p < 0.0001). In patients with SS and HIV/AIDS, presence of opportunistic infection did not significantly affect ICU admission rates (38% without and 36% with opportunistic infection; p = 0.0694) or survival (29% with or without opportunistic infection). When adjusted for age, gender, other comorbidities, race, infection site, payer type, failing organ systems, presence of opportunistic infection, hospital teaching status, and either ICU admission (only in mortality model) or mortality (only in ICU admission model), patients with SS and HIV/AIDS were more likely to die (OR (95% CI) = 2.41 (2.23–2.61)) compared with those without HIV/AIDS and were also significantly less likely to be admitted to the ICU (OR (95% CI) = 0.54 (0.51–0.59)).

We assessed adjusted mortality and ICU admission rates for patients with SS and comorbidities other than HIV/AIDS. When compared with patients with SS and HIV/AIDS only (i.e., no other comorbidities other than HIV/AIDS), patients with SS and no cormorbidities (OR (95% CI) = 0.36 (0.33–0.39)), or only diabetes (OR (95% CI) = 0.37 (0.33–0.42)), pulmonary disease (OR (95% CI) = 0.38 (0.33–0.43)), cardiovascular disease (OR (95% CI) = 0.39 (0.33–0.47)), or renal disease (OR (95% CI) = 0.67 (0.56–0.80)) were significantly less likely to die (Table 3). Those with SS and only liver disease (OR (95% CI) = 1.28 (1.14–1.44)), only neoplasm (OR (95% CI) = 1.79 (1.61–1.98)), or HIV with other comorbid illnesses (OR (95% CI) = 1.67 (1.47–1.90)) were more likely to die than those with SS and HIV/AIDS only. However, patients with SS without HIV/AIDS were universally more likely to be admitted to the ICU than patients with SS with HIV/AIDS regardless of their comorbidities (and associated mortality rate). In an additional comparison, we compared adjusted mortality and ICU admission rates between those with SS and HIV/AIDS and those with SS and metastatic cancer. When compared with those with SS and HIV/AIDS only, those with SS and metastatic cancer only were significantly more likely to die (OR (95% CI) = 2.29 (2.03–2.58)) and were also significantly more likely to be admitted to the ICU (OR (95% CI) = 1.41 (1.26–1.86)).

### 'Matched' analyses

To assess the robustness of our findings, we performed additional analyses in a subset of 'matched' patients. When we limited the analysis to a subset of patients aged 41 to 60 years, without comorbidities (other than HIV for those with HIV infection), covered by Medicaid or Medicare, who were admitted to a medical service in a teaching hospital, we obtained similar results to the results from the whole cohort whether we assessed patients who had respiratory infections (without opportunistic infections) or whether we looked only at those with opportunistic infections. In the 'matched' cohort with respiratory infections, those with HIV had, on average, significantly less costly hospital stays ($2,659 less, p < 0.0001); had shorter hospital stays (1.7 days less, p < 0.0001); were more likely to die (OR (95% CI) = 1.86 (1.35–2.56)); and were less likely to be admitted to the ICU (OR (95% CI) = 0.47 (0.35–0.63)). When we focused only on those with opportunistic infections, those with HIV had, on average, significantly less costly hospital stays ($4,490 less, p < 0.0001); had shorter LOS (1.6 days less, p < 0.0001); and were less likely to be admitted to the ICU (OR (95% CI) = 0.38 (0.25–0.59)) despite similar likelihood of death (OR (95% CI) = 1.31 (0.82–2.08)).

## Discussion

In this HAART-era study, we found that patients with SS and HIV/AIDS overall had less costly hospitalizations, were less likely to be admitted to the ICU, and had a greater in-hospital mortality than those without HIV/AIDS. HIV/AIDS patients had similar LOS, lower hospitalization costs, and greater mortality than those without HIV/AIDS whether they lived, died, or were admitted to the ICU. However, for patients with SS not in the ICU, the trends were different. Specifically, those with HIV/AIDS had significantly longer LOS and had somewhat higher mean hospitalization costs (and continued higher mortality rates) than those without HIV/AIDS. We also found that when compared with those with SS and HIV/AIDS, patients with SS without HIV/AIDS were universally more likely to be admitted to the ICU, even when they had comorbid illnesses with equal or worse expected in-hospital mortality (e.g., metastatic cancer). Those results were robust with qualitatively similar results in univariate, multivariable, and subgroup analyses.

Despite having higher mortality rates, patients with SS and HIV/AIDS were significantly less likely to be admitted to the ICU than patients with SS without HIV/AIDS. Nicolau and colleagues [22] studied patients with *Pneumocystis carinii *pneumonia with and without HIV/AIDS and had similar findings. What is unclear and cannot be discerned from our data is whether that difference in care is inappropriate because of physician or healthcare system bias or whether the difference is appropriate and based on differences in patient preference (e.g., advanced directives) or clinical differences between patients with and without HIV/AIDS. Existing evidence suggests there may be clinical biases against aggressive treatment of patients with SS and HIV/AIDS [23-26]. In our analysis, for patients with SS who were not admitted to the ICU, one could argue that those with HIV/AIDS were 'sicker' than those without HIV/AIDS because they had longer LOS, higher mean hospitalization costs, and higher mortality (in contrast to the overall trends that showed that, in general, patients with HIV/AIDS had similar LOS and lower hospitalization costs) and should have had more ICU utilization. Sasse and Wachter and colleagues [24-26] speculated that there is clinical bias that stems from a conception of HIV as a 'terminal' condition with poor overall long-term survival resulting in a provider-imposed limitation on medical care. We performed a limited exploration of this explanation with our data. If systematic withholding of ICU admission was indeed happening based on expected survival, then patients in our database with other comorbid illnesses with equal or higher in-hospital mortality rates (i.e., metastatic cancer) could also have been expected to have lower ICU admission rates. However, patients with SS without HIV/AIDS were universally more likely to be admitted to the ICU regardless of their comorbidities and associated mortality (including those with metastatic cancer).

The explanation for differences in ICU use may also lie in patient preferences. Given the emphasis on advanced directives in patients with HIV/AIDS that began before the HAART era [27-34], it is likely that more patients with HIV/AIDS than without HIV may have their wishes known *vis-à-vis *aggressive care and, thus, may have had their care decelerated, decreasing the use of aggressive measures and increasing use of palliative measures like hospice. Nonetheless, physicians caring for in-patients with HIV/AIDS, as well as the patients themselves, should be made aware of improvements in outcomes for critically ill patients with HIV/AIDS before making decisions about withholding or withdrawing aggressive care [7-9].

In regards to our cost results, we suspected that the cost differences might be explained by the differences in ICU admission and mortality (i.e., patients with SS and HIV/AIDS may die quickly outside the ICU thus using less resources); however, the difference in cost persisted even after stratifying by mortality, and in fact, for those not admitted to the ICU, costs were similar for those with and without HIV/AIDS. Furthermore, the difference in cost persisted even in our adjusted analyses that accounted for additional issues such as LOS, comorbidities, and failing organ systems, as well as in our 'matched' subgroup analyses. Others have compared resource use between patients with and without HIV/AIDS [35-37]. In those studies, patients with HIV/AIDS had significantly higher overall resource use. However, we found only one study that compared resource use in patients with and without HIV but with a similar discharge diagnosis, *Pneumocystis carinii *pneumonia [22]. The results of that study were similar to our current study in that patients with HIV/AIDS were less likely to be admitted to the ICU and had lower overall hospital costs.

The major limitations of our study relate to the use of administrative data. The general issues with using administrative data for research have been well documented by others [38,39]. Specifically in our study, we could only define severe sepsis and HIV using ICD-9-CM codes, rather than by clinical, laboratory, or physiological parameters. In these administrative data, we were unable to discern differences in patient preferences and pathophysiology between those with SS with and without HIV/AIDS that likely exist and might thereby explain the differences we found in care, resource use, and mortality. Additionally, we were unable to discern HIV disease severity other than coexisting presence of opportunistic infection (there are not separate ICD-9-CM codes for HIV and AIDS) and we lacked the treatment (antiretroviral therapy) and laboratory staging (viral load and CD4 cell count) data that could also have provided insight into the differences we found. Furthermore, by using ICD-9-CM codes to identify severe sepsis, the temporal overlap between infection and organ dysfunction was not confirmed. However, we did use validated approaches for identifying both HIV [17] and severe sepsis [13], and our results are consistent with other clinical studies that report outcomes for patients with severe sepsis (or sepsis syndrome) and HIV/AIDS [7,8,13,40]. Finally, treatment, ICU utilization, and mortality expectations have evolved over time for patients with HIV/AIDS. Thus, studying a fluid situation at one period in time (1999) is not optimal, and more recent longitudinal data would be useful and should be pursued as future work.

Despite the limitations, our study has several notable strengths. First, our finding of less aggressive care (lower cost of hospitalization and less ICU care) were robust with consistent findings using different analysis assumptions and methodologies. Second, using a large, multi-state administrative database allows us to easily generate reliable estimates of outcomes obviating the need for a large multi-center study and permits examination of care patterns and resource use simultaneously. Furthermore, our study has more power and generalizability than the small, single-site studies that have provided much of the evidence base for care of critically ill patients with HIV/AIDS [7,8,40-44]. Lastly, our study has a broad perspective that is not limited in focus to only HIV patients [6-10,40-44] or to patients receiving care in the ICU [7,8,10,41-43] but, rather, includes all patients with severe sepsis regardless of site of care within the acute care hospital thus permitting examination and comparison of care and outcomes in patients with and without HIV/AIDS with similar serious disease processes.

## Conclusion

In conclusion, we found a difference in care and outcome for patients with SS and HIV/AIDS, in that they had less costly hospitalizations, were less likely to be admitted to the ICU, and had greater in-hospital mortality than those without HIV/AIDS. Further research is needed to examine whether that difference in care persists over time and if it is inappropriate because of physician or healthcare system bias or whether the difference is appropriate and based on differences in patient preference or clinical differences between patients with and without HIV/AIDS.

## Key messages

• 	We found that patients with SS and HIV/AIDS overall had less costly hospitalizations, were less likely to be admitted to the ICU, and had a greater in-hospital mortality than those without HIV/AIDS.

• 	HIV/AIDS patients had similar LOS, lower hospitalization costs, and greater mortality than those without HIV/AIDS whether they lived, died, or were admitted to the ICU.

• 	When compared with those with SS and HIV/AIDS, patients with SS without HIV/AIDS were universally more likely to be admitted to the ICU, even when they had comorbid illnesses with equal or worse expected in-hospital mortality (e.g., metastatic cancer).

• 	Further research is needed to examine whether that difference in care persists over time and if it is inappropriate because of physician or healthcare system bias or whether the difference is appropriate and based on differences in patient preference or clinical differences between patients with and without HIV/AIDS.

## Abbreviations

CI = confidence interval; HAART = highly active antiretroviral therapy; ICD-9-CM = International Classification of Diseases, 9th revision, Clinical Modification; ICU = intensive care unit; LOS = length of stay; OR = odds ratio; SS = severe sepsis.

## Competing interests

JAJ and LB are full-time employees of Eli Lilly and Company. JMM and WTL have received research funding from Eli Lilly and Company. JMM is currently employed at GlaxoSmithKline.

## Authors' contributions

JMM designed the study, performed the analyses, and drafted the manuscript. LB conceived the project, assisted with interpretation of the data, and critically reviewed and revised the manuscript for important intellectual content. MSY assisted with analysis and interpretation of the data, and critically reviewed and revised the manuscript for important intellectual content. WTL acquired the dataset, assisted with analyses, and critically reviewed and revised the manuscript for important intellectual content. JAJ assisted with interpretation of the data, and critically reviewed and revised the manuscript for important intellectual content. All authors read and approved the final manuscript.

## Figures and Tables

**Table 1:** Characteristics of patients with severe sepsis

Characteristic^a^	HIV/AIDS	No HIV/AIDS
Number of admissions	7,638	66,382
Age		
Mean (SD)	41.9 (8.4)	49.9 (11.3)
Sex		
Female (%)	34	46
Male (%)	66	54
Race		
White (%)	20	56
Black (%)	49	18
Hispanic (%)	14	7
Other or not reported (%)	17	19
Insurance		
Commercial (%)	16	42
Medicaid/Medicare (%)	48	20
Other (%)^b^	36	38
Admission type		
Medical (%)	88	69
Surgical (%)	12	31
Teaching hospital	76	61
Number of comorbidities^c^		
0 (%)	70	49
≥1 (%)	30	51
Organ system failures		
Respiratory (%)	35	47
Cardiac (%)	16	22
Hematologic (%)	37	25
Neurologic (%)	10	7
Renal (%)	28	24
Hepatic (%)	2	2
Number of organ system failures^d^		
0 (%)	0	0
1 (%)	78	78
2 (%)	17	17
≥3 (%)	5	5
Infection		
Respiratory (%)	45	42
Opportunistic (%)	53	9

^a^The p value for difference between patients with and without HIV/AIDS is ≤ 0.0001 for all characteristics unless otherwise noted. ^b^0.0001 < p ≤ 0.0500. ^c^Number excludes HIV/AIDS. ^d^p > 0.0500.

**Table 2:** Impact of HIV infection on length of stay and total cost of admission for patients with severe sepsis

Outcome	Impact of HIV/AIDS	P value	Adjusted impact of HIV/AIDS	P value
Length of stay (days)	-0.8	0.0669	-0.9^a^	0.0516
Hospitalization cost ($)	-6,155	<0.0001	-2,706^b^	<0.0001

^a^Adjusted for mortality, intensive care unit (ICU) admission, age, gender, comorbidities, race, infection site, payer, failing organ systems, presence of opportunistic infection, and hospital teaching status (adjusted R^2 ^= 0.11). ^b^Adjusted for mortality, ICU admission, length of stay, age, gender, comorbidities, race, infection site, payer, failing organ systems, presence of opportunistic infection, and hospital teaching status (adjusted R^2 ^= 0.64).

**Table 3:** Likelihood of death or ICU admission by comorbidity for patients with severe sepsis

Comorbidity	Adjusted odds ratio for mortality^a ^(95% confidence interval)	Adjusted odds ratio for ICU admission^b ^(95% confidence interval)
Only HIV/AIDS	Reference group	Reference group
No comorbidity	0.36 (0.33–0.39)	1.85 (1.70–2.01)
Only diabetes	0.37 (0.33–0.42)	1.82 (1.66–2.00)
Only pulmonary disease	0.38 (0.33–0.43)	1.93 (1.71–2.18)
Only cardiovascular disease	0.39 (0.33–0.47)	1.93 (1.66–2.24)
Only renal disease	0.67 (0.56–0.80)	1.76 (1.52–2.04)
Only liver disease	1.28 (1.14–1.44)	2.40 (2.16–2.67)
Only neoplasm	1.79 (1.61–1.98)	1.62 (1.48–1.79)
HIV with other comorbid illness(es)	1.67 (1.47–1.90)	1.07 (0.95–1.22)
Multiple comorbid illnesses without HIV/AIDS	0.99 (0.89–1.10)	1.75 (1.59–1.93)

^a^Adjusted for intensive care unit (ICU) admission, age, gender, race, infection site, payer, failing organ systems, presence of opportunistic infection, and hospital teaching status (c-statistic = 0.80). ^b^Adjusted for mortality, age, gender, race, infection site, payer, failing organ systems, presence of opportunistic infection, and hospital teaching status (c-statistic = 0.79).
